# Construction of CD19 targeted dual- and enhanced dual-antibodies and their efficiency in the treatment of B cell malignancy

**DOI:** 10.1186/s40164-023-00423-0

**Published:** 2023-07-24

**Authors:** Manling Chen, Xiaoyu Liu, Nan Peng, Ting Zhang, Junli Mou, Huizhen He, Ying Wang, Yingxi Xu, Haiyan Xing, Kejing Tang, Zheng Tian, Qing Rao, Runxia Gu, Shaowei Qiu, Min Wang, Jianxiang Wang

**Affiliations:** 1 State Key Laboratory of Experimental Hematology, National Clinical Research Center for Blood Diseases, Tianjin Key Laboratory of Cell Therapy for Blood Diseases, Haihe Laboratory of Cell Ecosystem, Institute of Hematology & Blood Diseases Hospital, Chinese Academy of Medical Sciences & Peking Union Medical College, 288 Nanjing Road, Tianjin, 300020 China; 2 Tianjin Institutes of Health Science, Tianjin, 300020 China; 3 Present Address: National Clinical Research Center for Hematologic Disease, Peking University People’s Hospital, Peking University Institute of Hematology, Beijing, China; 4 Present Address: Department of Hematology, Nanfang Hospital, Southern Medical University, Guangzhou, China

**Keywords:** CD19, B-ALL, Dual-specific antibody, Immunotherapy

## Abstract

**Background:**

T cell-redirecting bispecific antibodies establish a connection between endogenous T cells and tumor cells, activating T cells function to eliminate tumor cells without ex vivo genetic alteration or manipulation. Here, we developed a novel dual-specific antibody (DuAb) and an enhanced DuAb (EDuAb) with different stimulation signal to activate T cells, and evaluated their impact on the treatment of acute lymphoblastic leukemia (ALL).

**Methods:**

The expression plasmids of the DuAb and EDuAb containing CD80 molecule were constructed by cloning heavy chain and light chain variable fragments from anti-human CD19 (HI19a) and CD3 (HIT3a) monoclonal antibody hybridomas, respectively. The activation and the anti-tumor efficacy of human T cells mediated by DuAb and EDuAb were evaluated in vitro. B-cell ALL xenograft NSG mouse model was established to investigate the therapeutic effect in vivo.

**Results:**

EDuAb promoted the optimal expansion of primary human T cells with low expression of inhibitory markers in vitro than DuAb did. Both DuAb and EDuAb showed a similar capability in inducing healthy donor T cells to specifically eliminate B-ALL cell lines and primary blasts from patients*.* The similar ability was also observed in the patient-derived T cells. In vivo study showed that both DuAb and EDuAb significantly alleviated tumor burden and extended survival of B-ALL xenograft NSG mice. The median survival of PBS, DuAb and EDuAb treatment groups were 27, 38 and 45 days, respectively. The phenotype of T cells and cytokine release in peripheral blood (PB) of B-ALL xenograft NSG mice on day 24 were analyzed as well. The results showed that the proportion of CD8^+^ T cells and cytokine levels, including IL-2, IFN-γ and TNF-α, were higher in the EDuAb group than that of DuAb. Moreover, both DuAb and EDuAb significantly decreased the residual leukemia cells in PB of B-ALL xenograft NSG mice.

**Conclusions:**

Both DuAb and EDuAb showed great potential as novel treatments for B-ALL in clinical applications. However, compared to DuAb, EDuAb showed a significant advantage in promoting the proliferation and survival of T cells. Furthermore, EDuAb showed a better promising effect on eliminating tumor cells and extending survival in vivo, which provides new insights for the development of new multi-specific antibodies.

**Supplementary Information:**

The online version contains supplementary material available at 10.1186/s40164-023-00423-0.

## Introduction

Acute lymphoblastic leukemia (ALL) is a hematological malignancy characterized by the development of immature lymphocytes. Disease-risk stratification and intensified chemotherapy protocols have significantly improved the outcome of patients with ALL, particularly in children and adolescents/young adults. However, the prognosis for older adults (≥ 40 years) and patients with relapsed or refractory ALL remains poor [1]. Allogeneic stem cell transplantation (allo-SCT) is a curative option for patients; however, the post-transplant mortality, morbidity and recurrence rate remain high and most patients do not proceed to allo-SCT [2–6]. Therefore, there remains a high unmet medical need for more effective immunotherapeutic options for ALL patients, especially who are resistant to current therapies.

Due to the wide expression of CD19 in normal B cells as well as B cell-derived leukemia and lymphomas, there has been rapid development of immunotherapy strategies targeting this antigen, including bispecific T cell engagers (BiTEs) and chimeric antigen receptor (CAR) T-cells. Even though CD19 CAR-T cells have achieved unprecedented clinical responses for B cell malignancies [7, 8], it still encounters with substantial challenges, such as expensive cost and long preparation time. However, T cell-redirecting bispecific antibodies are able to establish the connection between endogenous T cells and tumor cells in order to activate T cells function to eliminate cancer cells without the need for ex vivo genetic alteration or manipulation of the T cells. It can provide an off-the-shelf immune-oncology therapy. Many bispecific antibodies designed targeting special antigen for solid and hematologic malignancies are undergoing evaluation in preclinical experiments and clinical studies [9, 10]. The therapeutic potential of this approach is exemplified by Blinatumomab (Blincyto), a bispecific T cell engager (BiTE) targeting CD19 which showed significantly longer OS than chemotherapy group in relapsed or refractory B-cell precursor ALL [11]. Recently, other clinical trials also showed the well tolerated and effective treatment in elder patients and adults with minimal residual disease [12, 13]. Even so, the effect of bispecific antibody could still be improved to some extent.

Full T cell responsiveness depends on costimulatory signals triggered by the binding of B7 family ligands to CD28 receptor on T cells. The TCR and costimulatory signals act synergistically to induce the transcriptional activation of IL-2 and Bcl-X_L_ of NF-κB family, which are key response markers promoting T cells proliferation and survival [14, 15]. Consequently, anti-CD28 variable fragment was used in bispecific or trispecific antibodies to provide T cells the costimulatory signal in some studies [16–18]. Even though CD28 signal indeed enhanced fusion protein antibodies in pre-clinical research, we cannot ignore the lessons from the CD28 super agonist TGN1412 trial, in which six volunteers suffered life-threatening cytokine-release syndrome (CRS) [19, 20]. As a member of B7 family, CD80 has been demonstrated to have the function of costimulatory molecules in a large number of studies. Our previous studies also showed that upregulating CD80 expression on tumor cells or combining with bispecific antibody could enhance T cell function of lysing tumor cell [21–23]. Therefore, CD80, as the natural ligand of CD28, showed a good potential of enhancing the function of bispecific antibody.

In this study, we developed the novel dual-specific antibody (DuAb) and enhanced DuAb (EDuAb) with different stimulation signal to activate T cells and explored their effect on the treatment of B-ALL. Through evaluating their potential efficacy for B cell malignancy in vitro and in vivo, results showed that both DuAb and EDuAb had promising effect to eliminate tumor cells, and EDuAb showed a better effect to extend survival in vivo*,* which may represent a candidate strategy for B cell malignancy therapy and provide a new insight for constructing new multi-specific antibodies.

## Materials and methods

### Cell lines and human samples

Nalm6, K562, Namalwa cell lines used in this study were purchased from American Type Culture Collection (ATCC). CD19 knockout Nalm6 cell line (Nalm6^CD19KO^), luciferase transfected Nalm6 cell line (Nalm6^luciferase^) and CD19 overexpression K562 (K562^CD19^) cell line were constructed previously and preserved in our lab. All the tumor cell lines were grown in RPMI-1640 with 10% fetal bovine serum (FBS) at 37 °C with 5% CO_2_.

Blood samples of B-ALL patients admitted to Institute of Hematology & Blood Diseases Hospital, Chinese Academy of Medical Sciences & Peking Union Medical College (CAMS & PUMC), were collected and cultured in IMDM supplemented with 15% FBS, 100 ng/ml rhFLT3-L, 100 ng/ml rhSCF and 50 ng/ml rhTPO (PeproTech, USA).

Human T cells from healthy donors were isolated using Human T Cells Enrichment Cocktail (STEMCELL, USA) according to the manufacturer’s protocol. Patient derived T cells were isolated from B-ALL patients and cultured in lymphocyte medium KBM581 (Corning, USA) supplemented with 10% FBS and 50 IU/mL human interleukin-2 (IL-2) (R&D systems, USA).

### Construction, production and purification of DuAb and EDuAb

The anti-human CD19 scFv and anti-human CD3 scFv were derived respectively from HIB19a and HIT3a monoclonal antibody hybridoma clones in our previous studies [24–26]. CD80 fragment was synthesized according to NCBI GenBank (the amino acid sequence of the extracellular domain of the CD80 molecule: VIHVTKEVKEVATLSCGHNVSVEELAQTRIYWQKEKKMVLTMMSGDMNIWPEYKNRTIFDITNNLSIVILALRPSDEGTYECVVLKYEKDAFKREHLAEVTLSVKADFPTPSISDFEIPTSNIRRIICSTSGGFPEPHLSWLENGEELNAINTTVSQDPETELYAVSSKLDFNMTTNHSFMCLIKYGHLRVNQTFNWNTTKQEHFPDN). Gene fragments with different linkers were subcloned into the mammalian expression vector pcDNA 3.4 (Thermo Fisher Scientific, USA), and N-terminal signal peptide, His6-tag and C-terminal Strep tagII coding sequences were added to facilitate antibody purification and detection.

ExpiCHO-S cells (Gibco, USA) were transfected with pcDNA 3.4-DuAb or pcDNA 3.4-EDuAb using ExpiFectamine CHO Transfection Kit (Thermo Fisher Scientific, USA) according to the manufacturer’s protocol. The supernatant was collected and purified by StrepTrap XT excel column (Cytiva, USA) according to the manufacturer’s instruction. The antibodies were concentrated and buffer was changed using centrifugal filter devices (Millipore, USA). The antibodies were then aliquoted and stored at − 80 °C.

All antibodies were verified by Western blot analysis and quantified using His-tag ELISA detection kit (Genscript, USA). SDS-PAGE was used to monitor protein purification.

### Flow cytometry analysis

Flow cytometry analysis was performed on FACS CantoII (BD, Biosciences, USA) or Novo Cyte 2060R (Agilent, USA) and analyzed by FlowJo 10.4 software. The antibodies used in this study were purchased from Biolegend, USA, including anti-CD19 (Clone HIB19a), anti-CD3 (Clone HIT3a), antiCD107a (Clone H4A3), anti-CD4 (Clone RPA-T4), anti-CD8 (Clone SK1), anti-CD25 (Clone BC96), Anti-CD69 (Clone FN50), Anti-LAG3 (Clone 11C3C65), Anti-TIM3 (Clone F38-2E2), Anti-PD-1 (Clone EH12.2H7). Additionally, anti-BCL-X_L_ (Cell Signaling Technology, USA), anti-His-tag mAb (MBL, China), and Annexin V (Biolegend, USA) were used in this study.

For cell surface markers detection, cells were collected and washed with PBS once. Subsequently, the cells were incubated with antibody for 30 min at 4 °C in the dark, followed by a single wash prior to analysis.

The absolute cell count was analyzed by flow cytometry using CountBright Absolute Counting Beads (Thermo Fisher Scientific, USA). To detect cell apoptosis, Annexin V and PI were used according to the manufacturer's instructions.

### Avidity determination of DuAb and EDuAb

The human CD3-positive T cell line Jurkat cells and CD19-positive B cell line Nalm6 cells were used for binding activity analysis of DuAb and EDuAb by flow cytometry. Briefly, 1 × 10^5^ cells in 100 μL PBS were incubated with serial dilutions of DuAb or EDuAb for 30 min at room temperature. After washing twice with PBS, cell-bound DuAb or EDuAb was detected with APC-conjugated anti-His tag antibody. The mean fluorescence intensity (MFI) of cells was measured and analyzed with GraphPad Prism.

### Capture of kinetics

To determine the binding kinetics and affinity constants of DuAb and EDuAb with CD3 and CD19 proteins, we used an optical biosensor based on surface plasmon resonance (SPR) detection. A Biacore 8 K SPR system (Cytiva, USA) equipped with series S Sensor Chip CM5 was used to generate binding kinetic rate and affinity constants at 25 °C in a running buffer. The CM5 chip was activated and coated with human CD3E & CD3D heterodimer protein (Acro, China) and human CD19 protein (Acro, China) to detect the reactions between DuAb or EDuAb with CD3 and CD19 proteins, respectively. We followed the detailed protocol provided by Acro company. The binding kinetics and affinity constants were then generated based on the data obtained from the SPR system.

### Competitive binding assay

Nalm6 and Jurkat cells were incubated with 10 nM DuAb or EDuAb and 10 nM commercial PE-CY7 conjugated anti-CD3 antibody or anti-CD19 antibody in PBS buffer for 30 min at room temperature. After washing twice with PBS, the mean fluorescence intensity (MFI) of cells was measured by flow cytometer.

### Detection of cytokines release

The co-culture supernatant and NSG mice blood plasma were collected and immediately stored at − 80 °C until further analysis. The concentrations of cytokines in the samples were quantified using BD Cytometric Bead Array (CBA) human Th1/Th2/Th17 Cytokine Kit (BD bioscience, USA) and ELISA kits specific for IL-2, tumor necrosis factor (TNF‐α) and interferon gamma (IFN‐γ) (R&D systems, USA), following the manufacturer's instructions.

In order to determine the source of cytokines, except for the T cell proliferation assay and patient primary cell cytotoxicity assay where 50 IU/mL IL-2 was added, other experiments such as T cell activation and tumor cell line cytotoxicity assays were performed without IL-2 supplementation in the co-culture systems.

### T cell activation and proliferation assays

Human primary T cells were co-cultured with tumor cells (E:T ratio of 2:1) in 96-well plates with increasing concentrations of DuAb or EDuAb ranging from 0.001 to 10 nM, respectively. The activation markers CD25 and CD69 on T cells were detected by flow cytometry. The supernatant was collected for cytokine detection.

For long-term T cell proliferation assays, human primary T cells were incubated with 1 nM DuAb or EDuAb at 37 °C. The culture medium was changed every 2–3 days, and the T cells density was maintained at 5 × 10^5^ to 2 × 10^6^ cells/ml. T cell proliferation was analyzed using counting beads.

### In vitro* cytotoxicity assays*

To perform the cytotoxicity assay, 1 × 10^5^ T cells were co-cultured with 5 × 10^4^ target tumor cells (E:T ratios of 2:1) at increasing concentration of DuAb or EDuAb in 96-well plate with 200 μl /well of T cell growth medium without IL-2. After co-culture for 48 h or 72 h, T cells were collected and analyzed by flow cytometry. Tumor cells were distinguished from T cells by either overexpressing GFP or RFP, or by labeling them with cell tracing dyes. The percentage of specific cell lysis was calculated using the formula [1-Experimental residual/Control residual] × 100%.

For imaging assay, 1 × 10^5^ T cells were co-cultured with 1 × 10^4^ Nalm6^luciferase^ cells in 96-well plate with 200 μl/well of T cells growth medium without IL-2. Increasing concentrations of DuAb or EDuAb were added, and after 24 h of co-culture, the cells were incubated with luciferin for 5 min and then imaged using Xenogen IVIS Spectrum (PerkinElmer, USA).

### In vivo* study*

Female NSG mice of 6–8 weeks were purchased from Institute of Laboratory Animal Sciences (CAMS&PUMC, China). On day 0, 1 × 10^5^ Nalm6^luciferase^ cells were intravenously inoculated into NSG mice, which were then randomly divided into PBS, DuAb and EDuAb treatment groups based on their weight. On day 1, 5 and 9, mice were transplanted with 5 × 10^6^ human T cells, which were expanded in vitro. Following T cell engraftment, 5 pmol of DuAb or EDuAb or the same volume of PBS were intravenously administered every day. After 2 weeks of leukemia cells inoculation, in vivo imaging of the mice was performed using Xenogen IVIS Spectrum, and the bioluminescence intensity of images were analyzed at different days using Living Image software. Body weight and survival were dynamically monitored. On day 24, the percentage of residual tumor cells, human T cells and cytokine levels released in peripheral blood was analyzed. Pathological assays of bone marrow, spleen, liver and kidney were performed to evaluate the infiltration of leukemic cells.

### Statical analysis

At least three replicates of each experiment were carried out in this study. Significance statistics were analyzed by Student’s t test, one-way ANOVA or two-way ANOVA, Kaplan–Meier methods and a log-rank test via GraphPad Prism 8. Statistical significance is indicated by the symbols: *, P < 0.05; **, P < 0.01; ***, P < 0.001; ****, P < 0.0001.

## Results

### Design, production, and binding specificity of DuAb and EDuAb

**Fig. 1:**
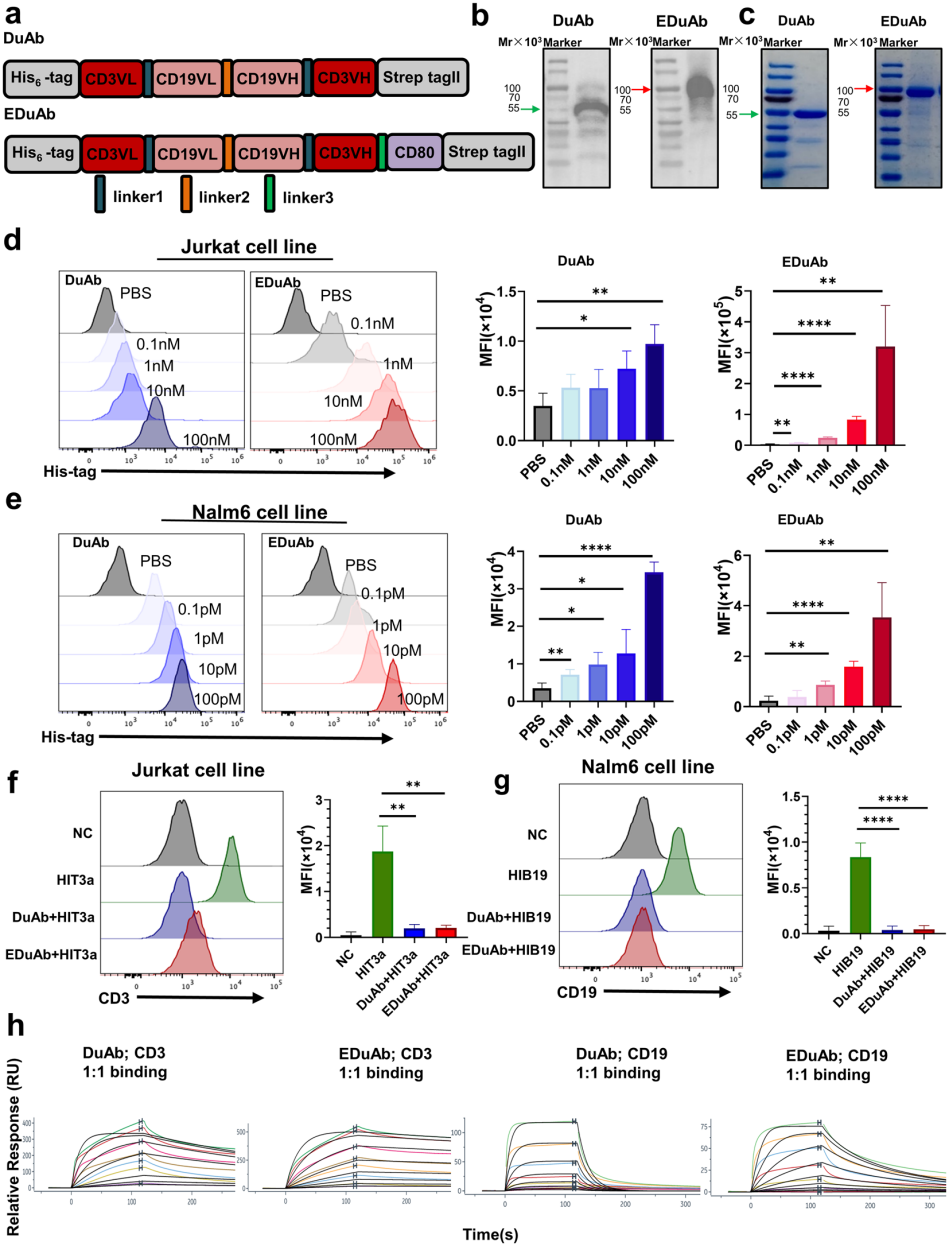
Design, production, and binding specificity of DuAb and EDuAb. **a** Schematic diagram of DuAb and EDuAb construct. **b** Western blot analysis of DuAb and EDuAb in supernatant of ExpiCHO-S cells transduced with expression plasmids. **c** Purified DuAb and EDuAb were analyzed by SDS-PAGE gel. **d**–**e** The binding affinity of DuAb and EDuAb to CD3^+^ and CD19^+^ cells were determined using flow cytometry (Left panel). MFI quantification and statistical analysis of the data (Right panel). **f**–**g** Competitive binding activity of DuAb and EDuAb with commercial PE-CY7-conjugated HIT3a and PE-CY7-conjugated HIB19 were detected using flow cytometry (Left panel). MFI quantification and statistical analysis of the data (Right panel). **h** The binding kinetics of DuAb or EDuAb to CD3 and CD19 protein were determined by Surface Plasmon Resonance (SPR)

DuAb was constructed using four antigen binding domains in a loop-like structure consisting of anti-human CD3 VL, anti-human CD19 VL, anti-human CD19 VH and anti-human CD3 VH, which were connected by different linker peptides (linker1: GGGGS; linker2: GSTSGSGKPGSGEGSTKG). EDuAb was established on the DuAb structure by adding CD80 extracellular domain to C-terminus via a long linker (linker3: GGGGSGGGGSGGGGSGGGGS) (Fig. 1a). To facilitate the detection and purification of the proteins, a hexa-histidine-tag (His_6_-tag) and strep-tag II (amino acid sequence: WSHPQFEK) were added to the N-terminus and C-terminus of the constructs, respectively. The fusion gene fragments of the constructs were cloned into the mammalian expression vector pcDNA 3.4 to develop pcDNA 3.4-DuAb and pcDNA 3.4-EDuAb, which were then transfected into the ExpiCHO-S cells for DuAb and EDuAb production.

The expressions of DuAb and EDuAb in the culture supernatant of ExpiCHO-S cells were detected by Western blot using anti-His tag antibody (Fig. 1b). Purified DuAb and EDuAb showed a high level of purity according to SDS-PAGE analysis (Fig. 1c). The calculated molecular weights of DuAb and EDuAb were 54.62KDa and 79.73KDa, respectively, consistent with the sizes detected in SDS-PAGE gel after purification.

Binding specificities of DuAb and EDuAb were confirmed through flow cytometry analysis of CD3^+^ Jurkat cells and CD19^+^ Nalm6 cells. After incubated with PBS or different concentration of DuAb and EDuAb, APC-conjugated anti-His tag antibody was used to detected the proportion of Jurkat cells and Nalm6 cells bound by DuAb and EDuAb. Results showed that the MFI of Jurkat and Nalm6 cells increased in a dose-dependent manner, indicating that DuAb and EDuAb could bind to CD3^+^ and CD19^+^ cells through specific interaction with CD3 and CD19 antigen (Fig. 1d, e). Furthermore, a competitive binding assay was performed by pre-blocking CD3 and CD19 antigens on Jurkat and Nalm6 cells with DuAb or EDuAb, followed by the addition of PE-CY7 conjugated commercial anti-CD3 (HIT3a clone) or anti-CD19 (HIB19a clone) antibodies. This led to the failed detection of PE-CY7 fluorescent signal, demonstrating that DuAb and EDuAb maintained the binding capacity of their derived HIT3a and HIB19a antibodies (Fig. 1f, g). The interaction and kinetic constant of DuAb or EDuAb with CD3 or CD19 proteins were examined by the Biacore 8 K System. The surface plasmon resonance (SPR) data analysis revealed that DuAb and EDuAb could bind with CD3 (CD3E&CD3D) and CD19 proteins in a dose-dependent manner (Fig. 1h). The KD value of DuAb and EDuAb binding to CD3 (CD3E&CD3D) was 43.9 nM and 38.3 nM, respectively, while that of CD19 was 380 nM and 64.5 nM, respectively. By means of SPR analysis results, EDuAb showed a similar affinity with CD3E&CD3D and a slightly better affinity with CD19 compared to DuAb (Table 1).

**Table 1:** The binding kinetics of DuAb or EDuAb to CD3 and CD19 protein

Method	Ligand	Analyte	Ka(1/Ms)	Kd(1/Ms)	KD(M)	Rmax(RU)	Fit Model
CM5	Human CD3E&CD3D hHeterodimer Protein	DuAb	3.14E + 05	2.63E-03	4.39E-08	402.1	1:1 binding
CM5	Human CD3E&CD3D hHeterodimer Protein	EDuAb	1.89E + 05	7.06E-04	3.82E-08	639.4	1:1 binding
CM5	Human CD19 Protein	DuAb	1.44E + 05	5.48E-02	3.80E-07	206.7	1:1 binding
CM5	Human CD19 Protein	EDuAb	2.55E + 05	1.65E-02	6.45E-08	85.3	1:1 binding

### The role of DuAb and EDuAb in activating T cells

**Fig. 2:**
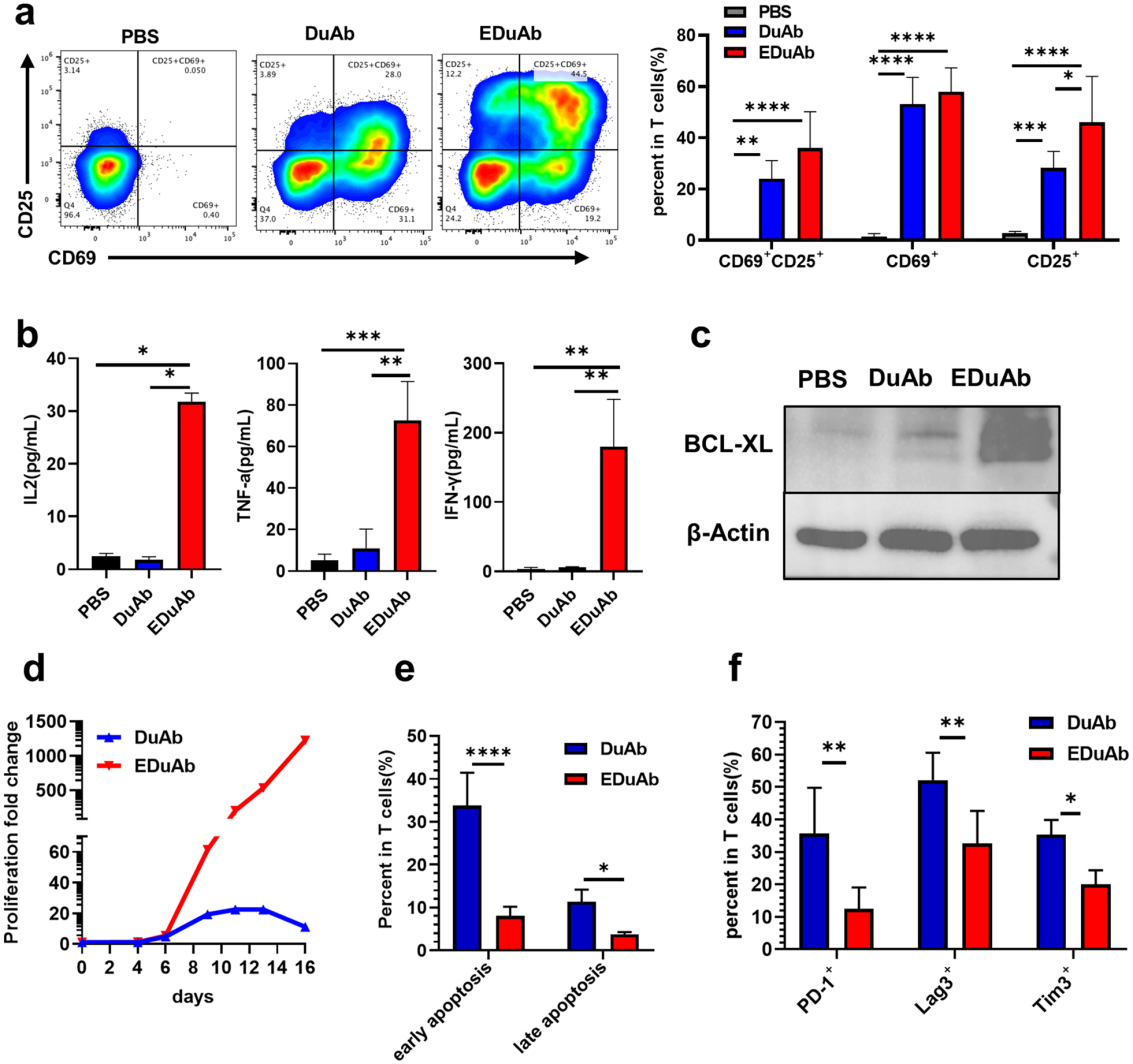
The role of DuAb and EDuAb in activating T cells. **a** Representative flow cytometry analysis of CD25 and CD69 on T cells after stimulated by DuAb and EDuAb (Left panel), quantification and statistical analysis of the data (Right panel). **b** Cytokines, including IL-2, TNF-α, IFN-γ, release from human primary T cells after stimulated by DuAb or EDuAb for 48 h. **c** Anti-apoptotic protein BCL-X_L_ was measured by Western blot. **d** Proliferation of T cells after stimulated by 1 nM DuAb or EDuAb. **e**, **f** Quantification of the apoptosis (early apoptosis: Annexin V^+^ PI^−^; late apoptosis: Annexin V^+^ PI^+^) and PD-1, Lag3 and Tim3 positive T cells after stimulated by DuAb and EDuAb in vitro

For optimal effector function and sustained proliferation of T cells, two signals are required. The receptor-CD3 complex signal induces transcriptional activation, leading to cytokine secretion, while the co-stimulation signal stimulates an alternative signal transduction pathway and inhibits programmed cell death. To determine whether DuAb, especially CD80 in EDuAb, could activate primary T cells, we evaluated the cytokine release level, activation and anti-apoptotic markers of primary T cells after incubated with PBS, DuAb or EDuAb at a concentration of 1 nM in human T cell culture medium with 10% FBS (no IL-2 was added in culture system). After culture for 48 h, the proportion of T cells expressing activated markers CD25 and CD69 was significantly higher in DuAb or EDuAb treated groups than that in PBS control group, and the proportion in the EDuAb treated group was higher than DuAb treated group (Fig. 2a). After culture for 72 h, the supernatant of co-culture system was collected and the cytokines release was detected. The results showed that there were higher cytokines release in EDuAb group than that of DuAb group, including IL-2, TNF-α and IFN-γ (Fig. 2b). EDuAb also stimulated primary T cells to upregulate anti-apoptotic protein BCL-X_L_ compared to DuAb (Fig. 2c). The above results indicated that the co-stimulation signal provided by CD80 molecules in EDuAb enhanced the activation of human primary T cells. To determine the effects of DuAb and EDuAb on T cell proliferation, DuAb or EDuAb were added to the primary T cell culture system with 10% FBS and 50 IU/mL IL-2 at a concentration of 1 nM, and the culture medium was changed to maintain T cell growing in proper density. Results showed that T cells activated by DuAb and EDuAb proliferated at almost the same rate in the first 6 days (fold changes on day 6 were 4.67 *vs* 5.33, *p* = 0.2358). After day 6, primary human T cells stimulated by EDuAb continued to proliferate, and the expansion folds reached 1220 times on day 16. However, T cells activated by DuAb stopped proliferating after day 9, and the peak of expansion folds remained at approximately 20 times from day 9 to day 13 (Fig. 2d). The inhibitory marker of T cells was also evaluated on day 9 of T cell proliferation assay in Fig. 2d. The results showed that compared to DuAb, EDuAb activated T cells expressed lower levels of apoptosis, PD-1, Lag3 and Tim3 (Fig. 2e, f). Taken together, these in vitro results demonstrate that EDuAb efficiently induces T cell activation, survival, and proliferation.

### ***DuAb and EDuAb mediate donor primary T cells lysis of CD19***^+^***cell lines ***in vitro

**Fig. 3:**
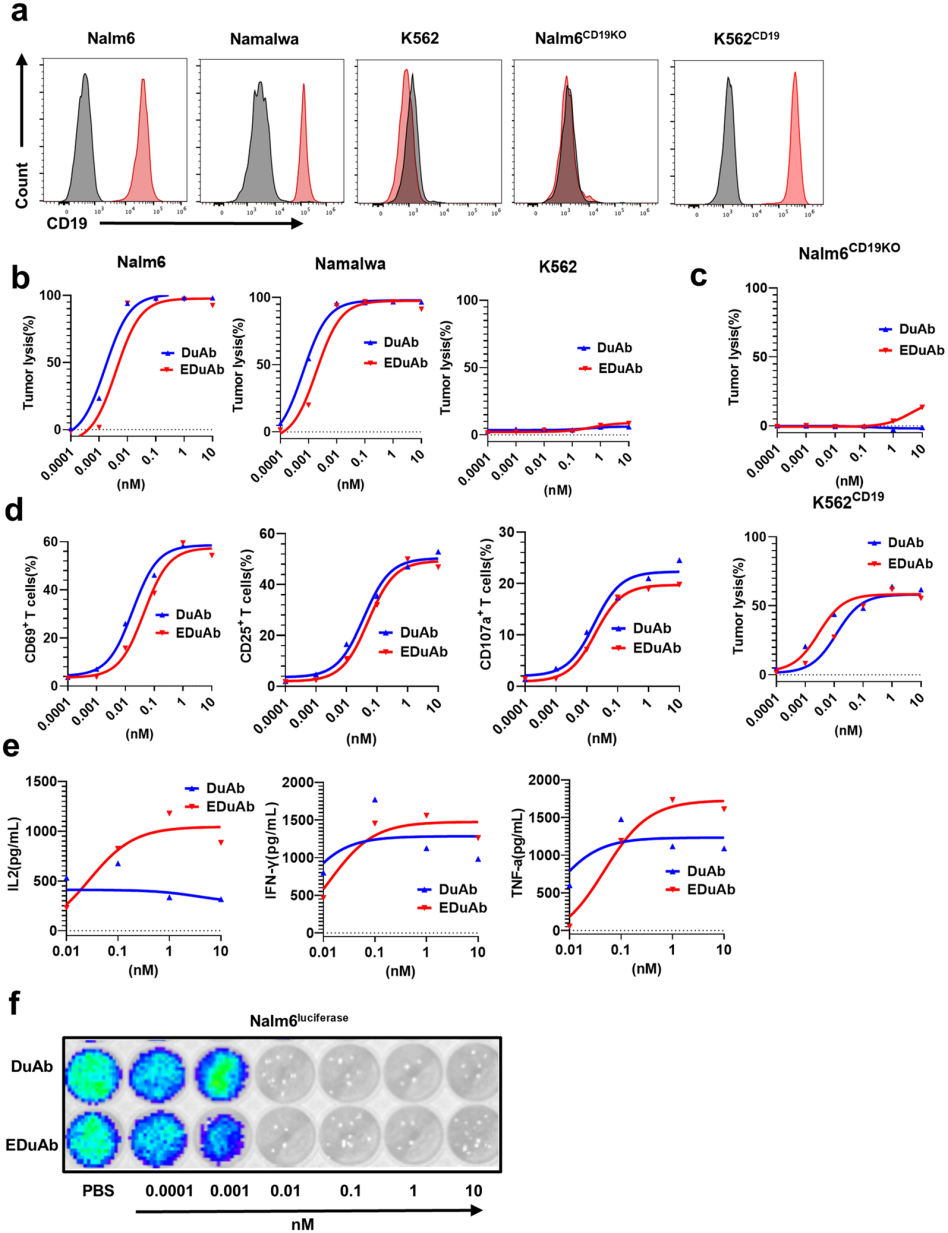
DuAb and EDuAb mediate donor primary T cells lysis of CD19 + cell lines in vitro. **a** Representative flow cytometry analysis showing the expression of CD19 in Nalm6, Namalwa, K562, Nalm6^CD19KO^ and K562^CD19^ cell lines. **b** Lysis of Nalm6, Namalwa and K562 cells after co-cultured with human primary T cells (E:T ratio of 2:1) in the presence of different concentrations of DuAb and EDuAb for 48 h. **c** Lysis of Nalm6^CD19KO^ (Upper panel) and K562^CD19^ (Lower panel) cells co-cultured with human primary T cells (E:T ratio of 2:1) in the presence of different concentrations of DuAb and EDuAb for 48 h. **d** Quantification and statistical analysis of CD69^+^, CD25^+^ and CD107a^+^ cells in human primary T cells after co-cultured with Nalm6 cells in the presence of different concentrations of DuAb or EDuAb. **e** Quantification of cytokines IL-2, TNF-α, and IFN-γ (from left to right panel) released into culture supernatants by T cells as results of activation and killing tumor cells mediated by DuAb or EDuAb. **f** Human primary T cells were co-cultured with Nalm6^luciferase^ cells (E:T ratio of 10:1) at increased concentrations of DuAb or EDuAb for 24 h. The bioluminescence image shown the residual of tumor cells in co-culture system

To determine the role of DuAb and EDuAb in specifically mediating CD19^+^ malignant cell lysis by human primary T cells, ALL or lymphoma cell lines (Nalm6, Namalwa) and CD19 negative control cell line (K562), were cocultured with human primary T cells in the presence of varying concentrations of DuAb and EDuAb. Nalm6^CD19KO^ and K562^CD19^ were also used in cytotoxicity assay to further verify the specific effects of DuAb and EDuAb on targeting CD19 antigen. The expression of CD19 on all the five cell lines were confirmed by flow cytometry (Fig. 3a). The cells were harvested and tested for residual malignant cells by flow cytometry after cocultured for 48 h at the E:T ratio of 2:1. CD19^+^ malignant cell lines, Nalm6 and Namalwa, were effectively killed in a dose-dependent manner, while K562 cells almost all survived from the killing of human primary T cells in the presence of DuAb and EDuAb (Fig. 3b). Similarly, DuAb and EDuAb couldn’t mediate T cells to kill Nalm6^CD19KO^ cells that did not express CD19, while K562^CD19^ cells that overexpressed CD19 could not survived from the killing of T lymphocytes mediated by them (Fig. 3c). The lysis efficiency is calculated as a relative value compared to that of the PBS group. The flow cytometry raw data and the residual ratio of tumor cells in the co-culture system are provided as supplementary information in Additional file 1: Fig S1. These results demonstrate that DuAb and EDuAb had specific cytotoxicity against CD19^+^ target cells.

In the presence of CD19-positive cells, both DuAb and EDuAb induced activation of T cell in a dose-dependent manner, which was reflected by upregulation of activation markers CD69 and CD25 and the degranulation level of T cells (Fig. 3d). The further hallmarks of T cell activation upon tumor lysis, levels of cytokines released in culture supernatants by T cells were measured, including IL-2, TNF-α and IFN-γ. Compared to DuAb, EDuAb induced a higher dose-dependent secretion of cytokines, especially IL-2 (Fig. 3e). These results suggested that human primary T cells were significantly activated in coculture system with CD19^+^ cells by DuAb and EDuAb, leading to the upregulation of activation markers, secretion of proinflammatory cytokines and killing of tumor cells.

Imaging assay also showed the specific role of DuAb and EDuAb mediating T cells lysis of Nalm6 cells (Fig. 3f). We also compared the function of DuAb with that of commercially available bispecific monoclonal antibody, Blincyto, which enables CD3^+^ T cells to recognize and eliminate CD19^+^ ALL blasts and has been approved for use in patients with relapsed or refractory B-cell precursor ALL [8]. Our results indicate that DuAb has similar effects with Blincyto in killing tumor cells (Additional file 1: Fig S2).

### DuAb and EDuAb mediate lysis of primary B-ALL cells by health donor T cells

**Fig. 4:**
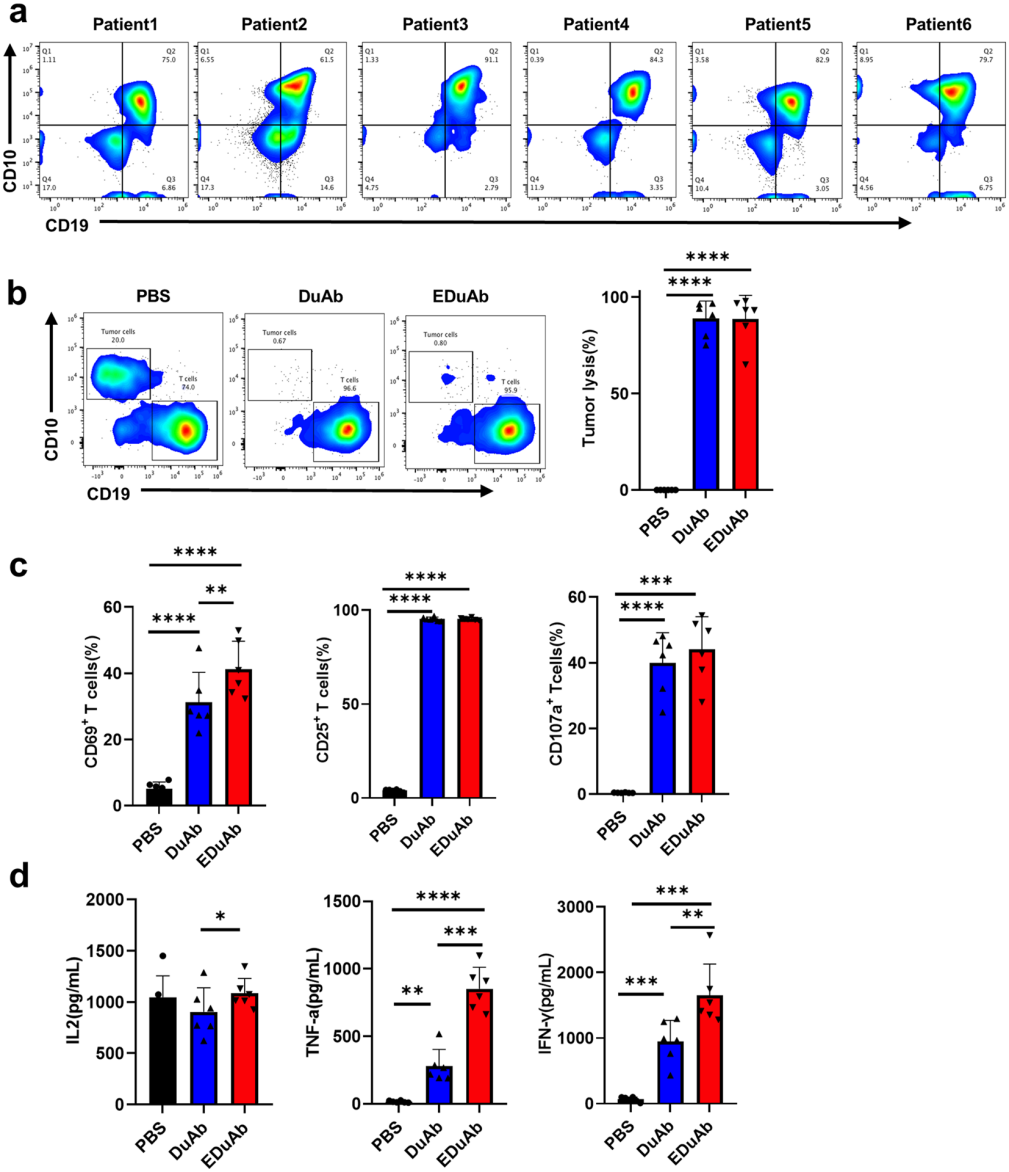
DuAb and EDuAb mediate lysis of primary B-ALL cells by health donor T cells. **a** Representative flow cytometry analysis of the expression of CD19 and CD10 on BMMNCs from six ALL patients. **b** Representative flow cytometry analysis of residual CD10^+^ tumor cells and remaining CD5^+^ T cells (Left panel). BMMNCs were incubated with health donor T cells (E:T ratio of 5:1) for 72 h at 1 nM concentration of DuAb or EDuAb. Tumor lysis was calculated based on residual CD10^+^ tumor cells (Right panel). (n = 6). **c** Quantification and statistical analysis of CD69^+^, CD25^+^ and CD107a^+^ cells in health donor T cells after cocultured with B-ALL cells in the presence of 1 nM DuAb or EDuAb. **d** Quantification of cytokines (IL-2, TNF-α, and IFN-γ) released into culture supernatants by health donor T cells as results of activation and killing tumor cells mediated by DuAb or EDuAb

The efficacy of DuAb and EDuAb was further tested on bone marrow mononuclear cells (BMMNC) from ALL patients. Among 6 patient samples (Table 2), the expressions of CD19 and CD10 were displayed by flow cytometry (Fig. 4a). After 72 h of co-culture, 1 nM of DuAb and EDuAb could successfully mediate healthy donor T cells killing of tumor cells at E:T ratio of 5:1. CD5 and CD10 were used to distinguish T cells from tumor cells in this study. Tumor lysis was calculated based on residual CD10^+^ tumor cells detected by flow cytometry (Fig. 4b). The upregulation of CD69 and CD25 activation markers, as well as the CD107a degranulation marker, were observed (Fig. 4c). Co-culture experiments also revealed that EDuAb induced T cells to secrete higher levels of cytokines than that of DuAb (Fig. 4d).

**Table 2:** Patient information

Patient ID	Disease	Sample type	CD19^+^CD10^+^ (%)
Patient1	ALL	BM	75
Patient2	ALL	BM	61.5
Patient3	ALL	BM	91.1
Patient4	ALL	BM	84.3
Patient5	ALL	BM	82.9
Patient6	ALL	BM	79.7

### DuAb and EDuAb induce the activation of T cells derived from all patients and mediate the killing of their own ALL cells.

**Fig. 5:**
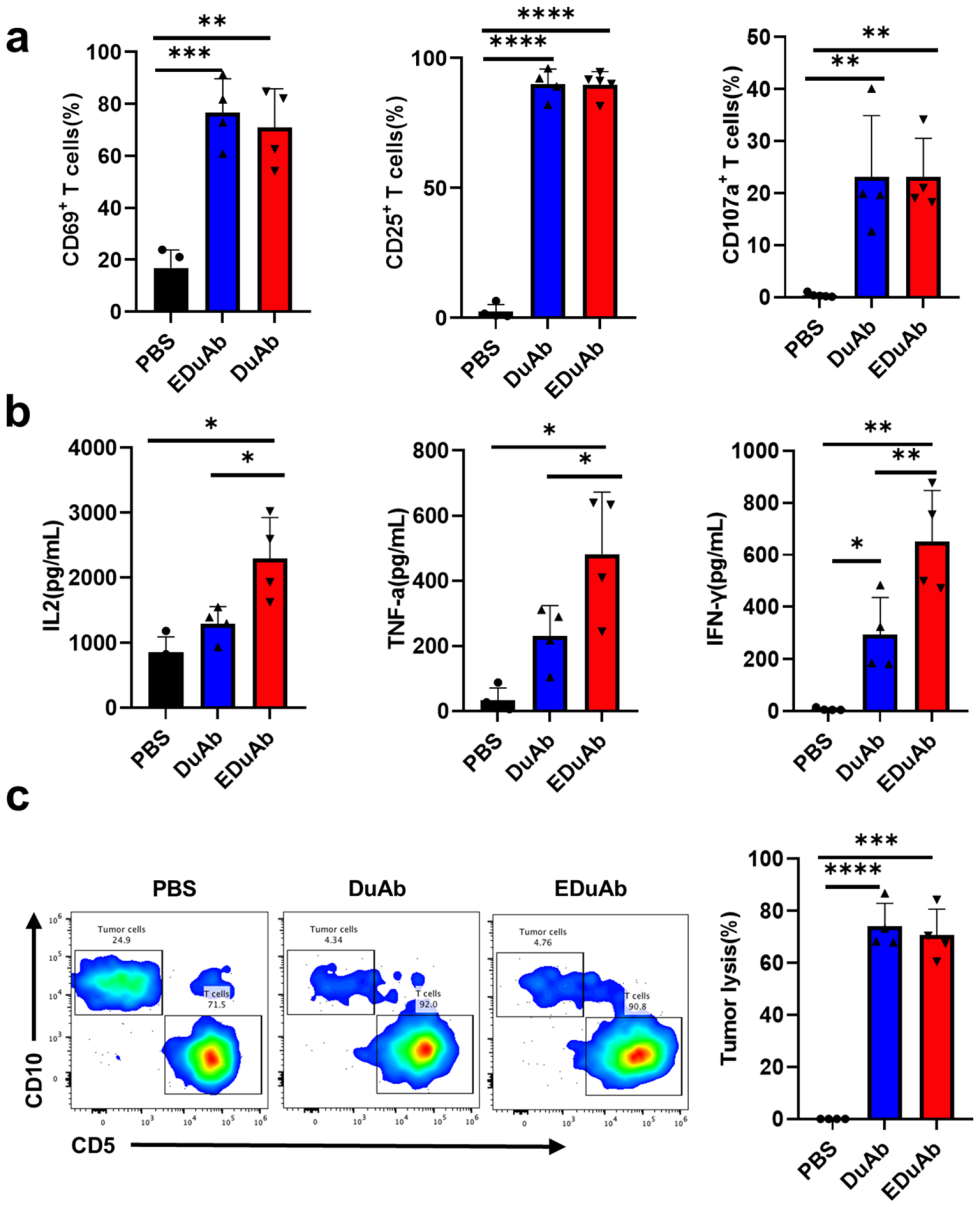
DuAb and EDuAb induce the activation of T cells derived from ALL patients and mediate the killing of their own ALL cells **a** Quantification and statistical analysis of CD69^+^, CD25^+^ and CD107a^+^ cells in T cells derived from ALL patients after co-cultured with their own ALL cells in the presence of DuAb or EDuAb. **b** Quantification of cytokines (IL-2, TNF-α, and IFN-γ) released into culture supernatants by patient derived T cells as results of activation and killing their own ALL cells mediated by DuAb or EDuAb. **c** Primary ALL cells were incubated with the ALL-derived T cells (E:T ratio of 5:1) for 72 h at 1 nM concentration of DuAb or EDuAb. Representative flow cytometry analysis of residual CD10^+^ tumor cells and remaining CD5^+^ T cells (Left panel). Tumor lysis was calculated based on residual CD10^+^ tumor cells (Right panel). (n = 4)

To test whether DuAb and EDuAb exposure could activate T cells from ALL patients and mediate the lysis of ALL cells, T cells and ALL tumor cells were sorted from ALL patient BMMNC samples and co-cultured with 1 nM DuAb or EDuAb at an E:T ratio of 5:1. Remarkably, addition of DuAb and EDuAb could induce the activation of T cells derived from ALL patients, shown by upregulation of activation markers CD69, CD25, the degranulation marker CD107a, and the production of IL-2, TNF-α and IFN-γ (Fig. 5a, b). In line with this, ALL-derived T cells could also efficiently lyse their own ALL tumor cells in presence of DuAb and EDuAb (Fig. 5c). Taken together, treatment with DuAb and EDuAb could induce functional activation of ALL derived-T cells and lead to potent lysis of ALL cells, which shed a light on the following clinical application.

### In vivo* activity of DuAb and EDuAb*

**Fig. 6:**
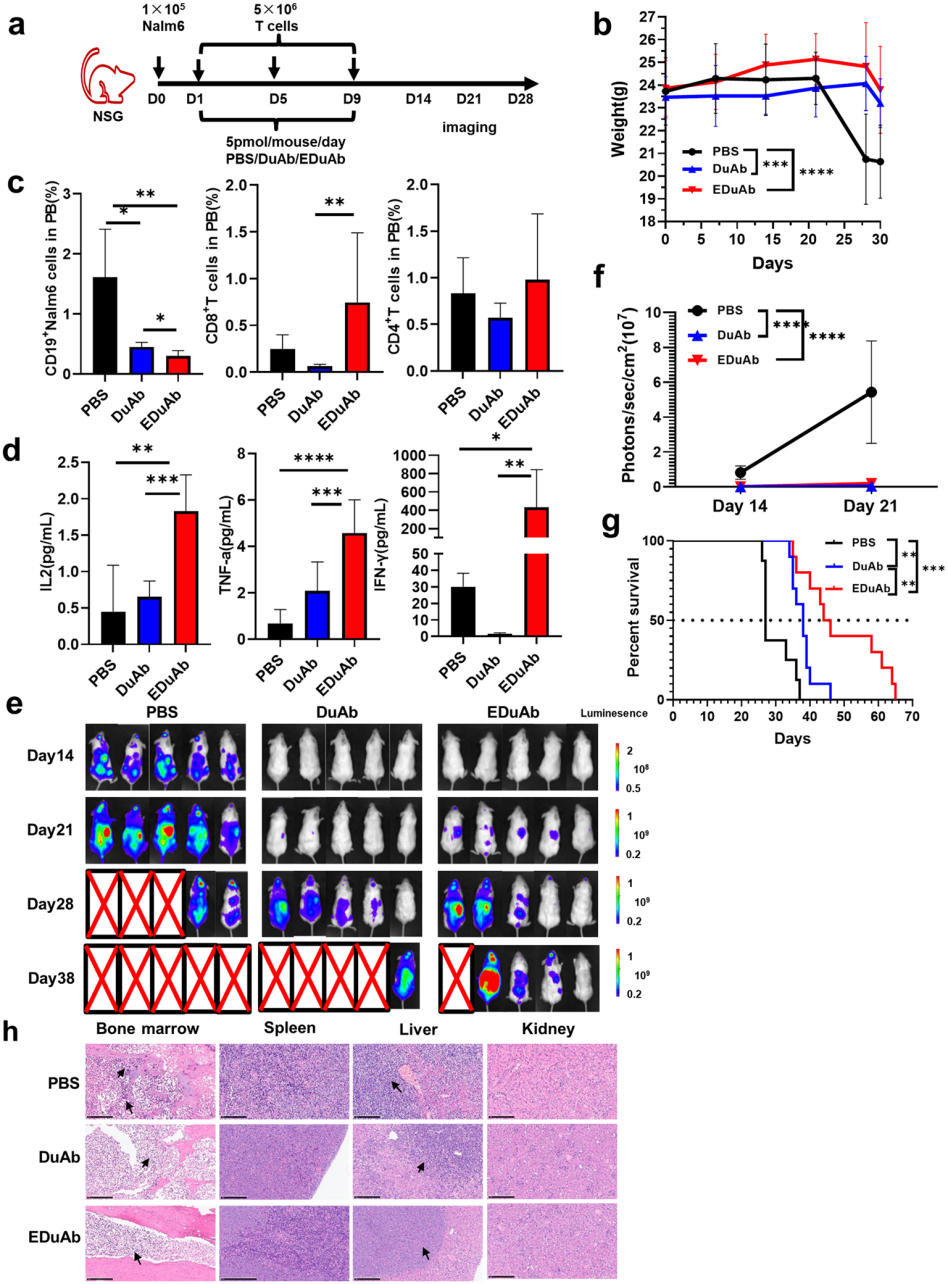
In vivo activity of DuAb and EDuAb **a** Schematic diagram of in vivo evaluation of DuAb and EDuAb. **b** Average body weight of three groups after PBS, DuAb and EDuAb treatment. **c** The residual Nalm6 cells (Left panel) and the phenotype of T cells (Middle and Right panel) in PB on day 24. **d** Cytokine release in PB on day24. **e** Representative IVIS imaging of tumor burden monitored by BLI at day 14, 21, 28 and 38. **f** Average radiance quantification (p/sec/cm^2^/sr) of image **e** for Nalm6 cells on day 14 and 21. **g** Kaplan–Meier survival curves of three groups. **h** Pathological analysis of the bone marrow, spleen, liver and kidney of the three representative mice in PBS, DuAb and EDuAb groups at their terminal stage by using HE staining

To evaluate the efficacy of DuAb and EDuAb in vivo, B-ALL xenograft NSG mice were established. The NSG mice were implanted with 1 × 10^5^ Nalm6 cells, then treated with 5 × 10^6^ human T cells expanded in vitro intravenously on day 1, 5 and 9. DuAb, EDuAb and PBS were administered once per day for a total 9 days at a dose of 5 pmol/mouse. The regimen of in vivo experiments was shown in Fig. 6a. Compared to the PBS group, no obvious loss of body weight was observed in DuAb and EDuAb group before day 28 (Fig. 6b). Both DuAb and EDuAb led to a significant reduction in residual Nalm6 cells in PB of B-ALL xenograft NSG mice, the residual tumor cells on day 24 were 1.61%, 0.45% and 0.29% in PBS, DuAb and EDuAb groups, respectively (PBS *vs* DuAb, *p* = 0.0134; PBS *vs* EDuAb, *p* = 0.0034; DuAb *vs* EDuAb, *p* = 0.0169). The phenotype of T cells and cytokine release in PB of B-ALL xenograft NSG mice on day 24 were analyzed as well. The results showed that the proportion of CD8^+^ T cells and the cytokine levels, including IL-2, TNF-α and IFN-γ, were higher in the EDuAb group than that of DuAb (CD8^+^ cells: 0.746% *vs* 0.064%, *p* = 0.0079; IL-2: 1.862 pg/ml *vs* 0.654 pg/ml, *p* = 0.0014; TNF-α: 4.57 pg/ml *vs* 2.08 pg/ml, *p* = 0.018; IFN-γ: 432.27 pg/ml *vs* 1.48 pg/ml, *p* = 0.047;) (Fig. 6c, d). The tumor burden in the mice was examined by bioluminescence imaging (BLI) on day 14, 21, 28 and 38 (Fig. 6e). The intensity of bioluminescence signal (photons/s/cm^2^) of DuAb and EDuAb group was obviously lower than that of PBS group (Fig. 6f). The median survival of PBS, DuAb and EDuAb treatment group were 27, 38 and 45 days, respectively, illustrating that EDuAb had a more effective therapeutic effect in vivo than that of DuAb (*p* = 0.0066) (Fig. 6g). To ensure accurate identification of leukemia-related causes of death, HE staining was performed to show the morphology of cells from all the mice at their terminal stage. Pathological analysis of the bone marrow, spleen, liver and kidney of the three representative mice in PBS, DuAb and EDuAb groups were shown in Fig. 6h.

## Discussion

The field of immunotherapy has achieved tremendous and encouraging progress over the past decade. Especially, the multi-specific antibody therapeutics have emerged as successful treatment options for complex illnesses, including checkpoint inhibition, antigen recognition of malignant cells, and molecular basis of T cell activation. Efficacy has also been observed in pre-clinical and clinical investigations using T cells engagers which could be an agent capable of inducing targeted T cell-mediated killing of the recognized tumor cells. In recent years, Blinatumomab, the first-in-class commercial bispecific antibody targeting CD19 and CD3, led to improved outcomes in the treatment of B cells malignancy [11, 27]. Despite these advances, there still have an emergence need to improve the efficacy of immunotherapy.

CD19, a membrane protein expressed on the surface of normal and most malignant B cells, is the most reliable cell-surface target in immunotherapies for B cell malignancies. However, the scFvs used in these studies were primarily derived from the hybridoma clones of FMC63 or SJ25C1, which may not provide long-term efficacy due to the loss of CD19 cognate epitope recognized by anti-CD19 scFv in case of the CD19-antigen mutations [28, 29]. To address this issue, alternative scFv binding to different CD19 epitopes may provide better treatment options for patients. In this study, we constructed DuAb and EDuAb by using a new scFv derived from clone HIB19a, which recognize a different binding epitope on the CD19 extracellular domain (ECD) than that of FCM63. In our previous work, CD19 CAR-T cells derived from clone HIB19a showed a high efficiency against malignant cells in patients [25, 26, 30]. In this study, DuAb and EDuAb also exhibited a specific ability to bind to the CD19 antigen.

Activation of T cells is critical for their immune response, and it requires two signals from CD3 and CD28 stimulation. The CD3 scFv used in DuAb and EDuAb provides T cells with the first signal, which derived from clone HIT3a previously developed in our institute. However, stimulation of the first signaling pathway alone, in the absence of the second signal, may led to activation-induced cell death and ultimately limit the anti-tumor response. Here, we used CD80 molecule to enhance the function of DuAb by binding to CD28 on T cells to provide the second signal and promote the survival of the activated T cells. Results in this study showed that EDuAb had a more robust effect on promoting the expansion of primary human T cells with low expression of inhibitor markers in vitro, compared to DuAb. Additionally, BCL-X_L_ was upregulated and IL-2 secretion was increased in EDuAb group. In vivo experiments also demonstrated that EDuAb had a more promising effect on eliminating tumor cells and extending survival compared to DuAb. These data showed that CD80 exactly enhanced DuAb function by providing the second signal.

Our previous study demonstrated that scFv function can be influenced by the type of linker used and the order of the heavy and light chain, even with the same nucleotide sequences [31]. Therefore, several linkers and different arrangements of CD3VL/CD3VL/CD19VL/CD19VH were evaluated to optimize the functionality of the dual-antibody in this study, while we only present the structures in which DuAb and EDuAb exhibit the best capacity in eliminating tumor cells. In comparison to the commercial CD19/CD3 bispecific antibody Blinatumomab, DuAb showed similar potential in mediating human primary T cells to eliminate tumor cells. Cytotoxicity and proliferation ability are two important factors that can evaluate the quality and anti-tumor efficacy of immune cells. Our findings suggested that DuAb and EDuAb have promising potential for clinical applications.

The efficacies of DuAb and EDuAb in humans have not yet to be evaluated, and the main issue should be addressed is the risk of CRS occurring in bispecific antibodies and CAR-T therapy, which can develop into fatal multiple organ failure in severe cases [32, 33]. In our study, we observed that mice treated by EDuAb had higher levels of peripheral T cells and circulating cytokines, including IL-2, TNF-α and IFN-γ on day 24. However, compared with DuAb group, there was no significant weight loss observed in EDuAb group, indicating that EDuAb regimens were safe in the mouse model. Before clinical application, further pre-clinical investigation and safety experiments, such as pharmacokinetics, toxicity and immune stimulation experiments in human primates (NHPs), are still required.

## Conclusions

In summary, this study shows that both DuAb and EDuAb hold great promise for clinical application as a novel treatment for B-ALL. Compared to DuAb, EDuAb exhibite significant advantages in promoting the proliferation and survival of T cells. Additionally, EDuAb demonstrate superior efficacy in eliminating tumor cells and prolonging survival in vivo, which provides new insights for constructing new multi-specific antibodies.

## Supplementary Information

**Additional file 1: Figure S1.** DuAb and EDuAb mediate donor primary T cells lysis of CD19+ cell lines in vitro (a-e) Representative flow cytometry analysis of the percentage of tumor cell residual in DuAb group and EDuAb group (Left panel), quantification analysis of the tumor cells residual at different concentrations of DuAb and EDuAb treatment (Right panel). **Figure S2.** Compared the function of DuAb with commercial bispecific monoclonal antibody (a) Representative flow cytometry analysis of the percentage of Nalm6 cell lysis in DuAb group and Blincyto group (Left panel), quantification and statistical analysis of the Nalm6 cells lysis at different concentrations of DuAb and Blincyto treatment (Right panel) (b) The proportion of CD69+, CD25+ and CD107a+ T cells after cocultured with Nalm6 cells at 1nM concentration of DuAb or Blincyto.

## Data Availability

The datasets used during the current study are available from the corresponding author on reasonable request.

## Abbreviations

DuAb: Dual-specific antibody
EDuAb: Enhanced dual-specific antibody
scFv: Single-chain variable fragment
ALL: Acute lymphoblastic leukemia
E: T: Effector to target
BMMNC: Bone marrow mononuclear cell
CAR: Chimeric antigen receptor
CRS: Cytokine release syndrome
allo-SCT: Allogeneic stem cell transplantation
MFI: Mean fluorescence intensity
CAMS & PUMC: Chinese Academy of Medical Sciences & Peking Union Medical College
