# Supplementary material for: Construction of CD19 targeted dual- and enhanced dual-antibodies and their efficiency in the treatment of B cell malignancy
Source: Exp Hematol Oncol. 2023 Jul 24;12:64. doi: 10.1186/s40164-023-00423-0 (PMC10367426; doi:10.1186/s40164-023-00423-0)
Supplement: Supplementary file 1 — Additional file 1: Figure S1. DuAb and EDuAb mediate donor primary T cells lysis of CD19+ cell lines in vitro (a-e) Representative flow cytometry analysis of the percentage of tumor cell residual in DuAb group and EDuAb group (Left panel), quantification analysis of the tumor cells residual at different concentrations of DuAb and EDuAb treatment (Right panel). Figure S2. Compared the function of DuAb with commercial bispecific monoclonal antibody (a) Representative flow cytometry analysis of the percentage of Nalm6 cell lysis in DuAb group and Blincyto group (Left panel), quantification and statistical analysis of the Nalm6 cells lysis at different concentrations of DuAb and Blincyto treatment (Right panel) (b) The proportion of CD69+, CD25+ and CD107a+ T cells after cocultured with Nalm6 cells at 1nM concentration of DuAb or Blincyto. [file 40164_2023_423_MOESM1_ESM.docx]

**Supplementary figures**

**
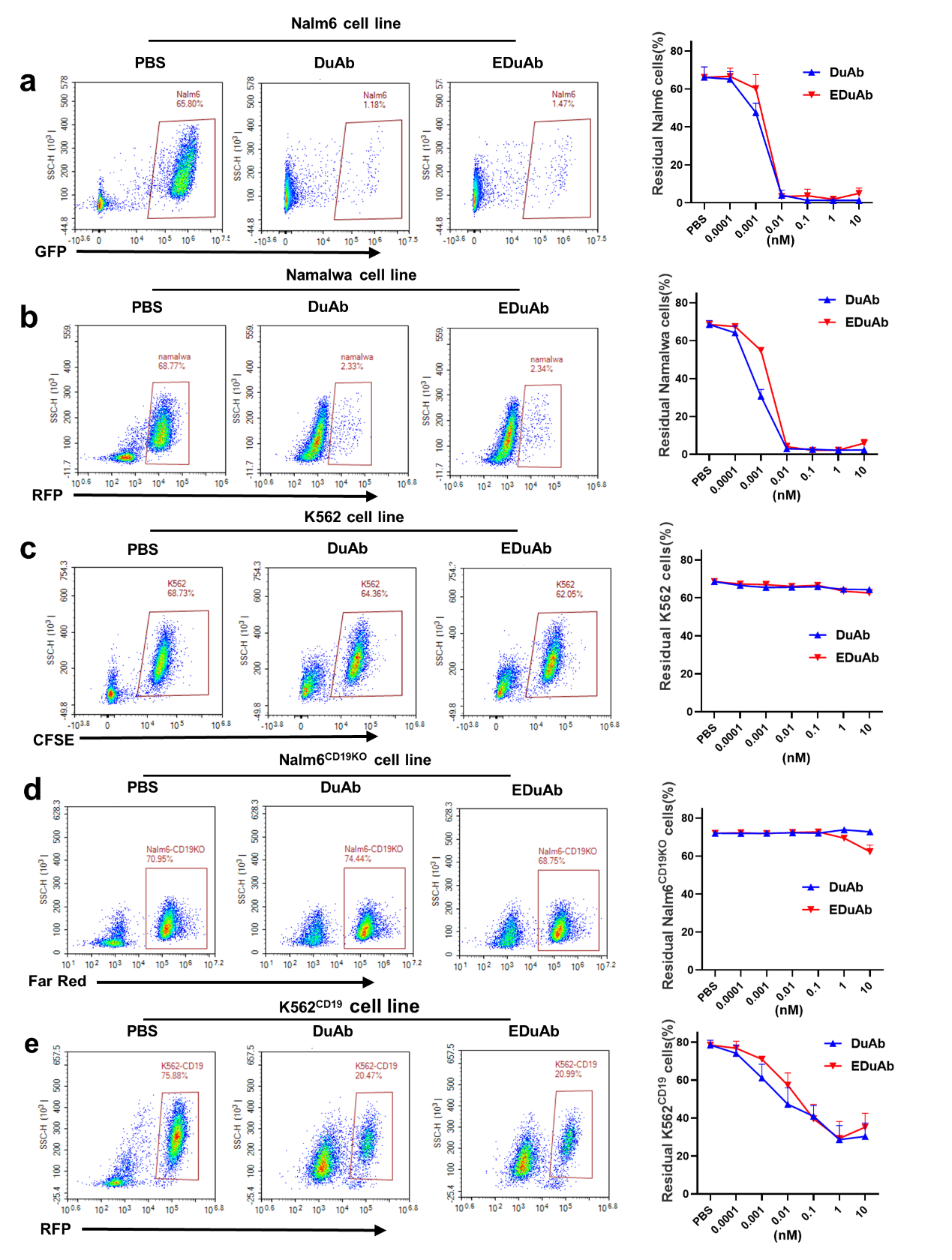
**

**Figure S1. DuAb and EDuAb mediate donor primary T cells lysis of CD19+ cell lines *in vitro*** **(a-e)** Representative flow cytometry analysis of the percentage of tumor cell residual in DuAb group and EDuAb group (Left panel), quantification analysis of the tumor cells residual at different concentrations of DuAb and EDuAb treatment (Right panel).

**
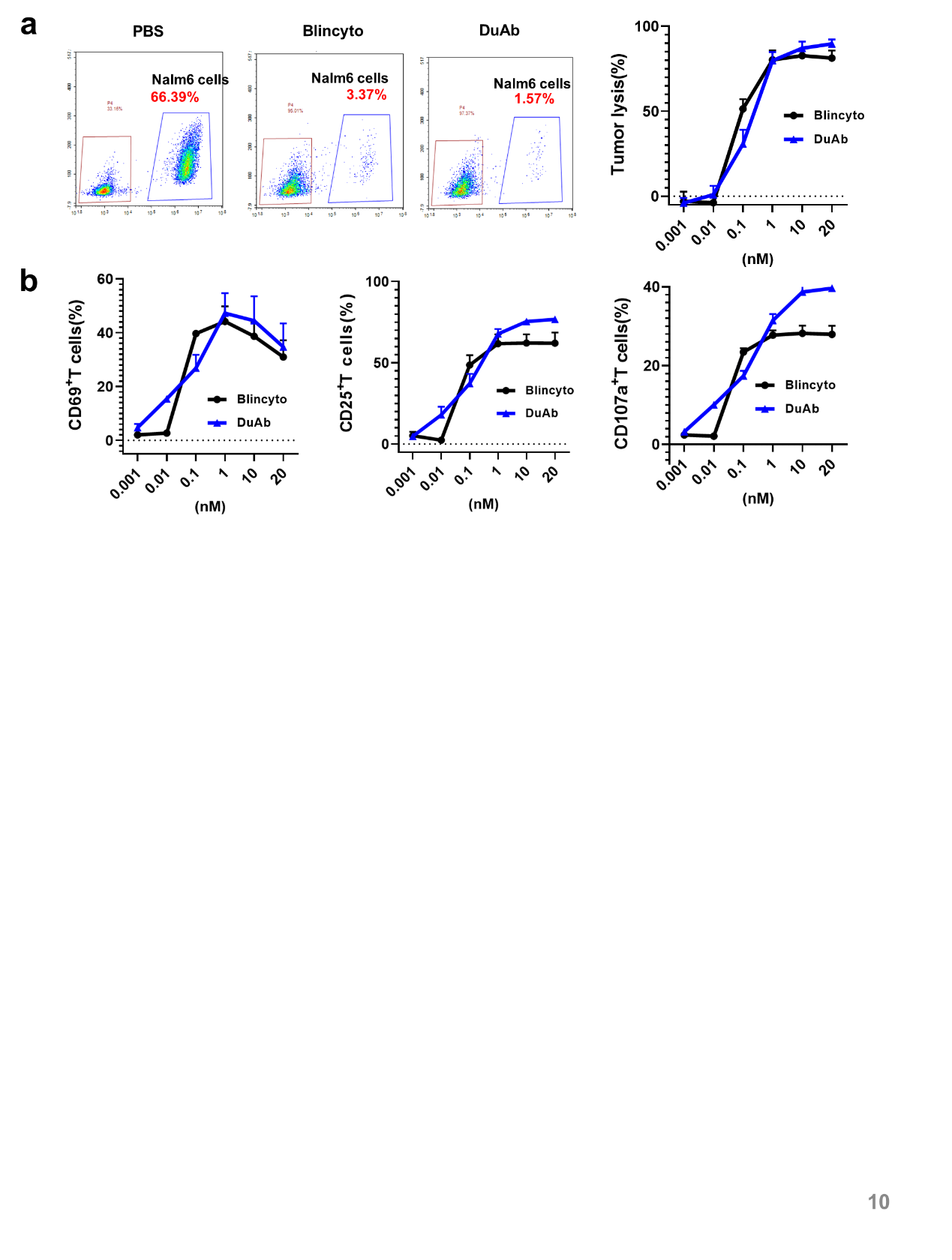
**

**Figure S2. Compared the function of DuAb with commercial bispecific monoclonal antibody (a)** Representative flow cytometry analysis of the percentage of Nalm6 cell lysis in DuAb group and Blincyto group (Left panel), quantification and statistical analysis of the Nalm6 cells lysis at different concentrations of DuAb and Blincyto treatment (Right panel) **(b)** The proportion of CD69^+^, CD25^+^ and CD107a^+^ T cells after cocultured with Nalm6 cells at 1nM concentration of DuAb or Blincyto.
